# Eye-Movements During Navigation in a Virtual Environment: Sex Differences and Relationship to Sex Hormones

**DOI:** 10.3389/fnins.2022.755393

**Published:** 2022-04-29

**Authors:** TiAnni Harris, Johanna Hagg, Belinda Pletzer

**Affiliations:** Department of Psychology and Centre for Cognitive Neuroscience, University of Salzburg, Salzburg, Austria

**Keywords:** spatial navigation, eye-tracking, virtual reality, sex differences, hormones, wayfinding

## Abstract

Sex differences in spatial navigation have been related to different navigation strategies. For example, women are more likely to utilize local landmark-information in the environment compared to men. Furthermore, sex differences appear to be more pronounced when distances need to be judged in Euclidian terms and an allocentric representation of the environment is necessary. This suggests differential attentional processes during spatial navigation in men and women. However, eye-tracking studies on spatial navigation exploring these attentional processes are rare. The present study (39 men and 36 women) set out to investigate sex differences in eye-movements during spatial navigation in a 3D environment using virtual reality goggles. While we observed the expected sex differences in overall navigation performance, women did not benefit from the landmark-based instructions. Gaze fixations were in accordance with the preferred Euclidian strategy in men, but did not confirm the expected landmark-based strategy in women. However, high estradiol levels where related to an increased focus on landmark information. Surprisingly, women showed longer gaze distances than men, although the utilization of distal landmarks has been related to allocentric representations preferred by men. In fact, larger gaze distances related to slower navigation, even though previous studies suggest that the utilization of distal landmarks is beneficial for navigation. The findings are discussed with respect to the utility of virtual reality presentation for studies on sex differences in navigation. While virtual reality allows a full first-person immersion in the environment, proprioceptive and vestibular information is lacking.

## Introduction

Scientific research on spatial navigation has progressed immensely over the past years. Different navigation tasks focus on different aspects of navigation, e.g., real-world navigation, virtual navigation, imagined navigation, spatial memory recall or viewing navigationally relevant stimuli (Epstein et al., 2017). Most commonly, navigation tasks focus on goal-directed navigation. Goal-directed navigation is defined as navigation through an environment toward a predetermined goal, or getting from point A (current location) to point B (goal) (Ekstrom et al., 2018). The current study focuses on sex differences in goal-directed navigation.

The process of navigation can be influenced by the properties of the environment itself, the navigator’s knowledge about the environment and the strategies employed by the navigator (Spiers and Barry, 2015). In experimental conditions it is possible to manipulate and control the environment, as well as the navigator’s knowledge about the environment, while the navigator’s strategy is often investigated. There are numerous definitions and categorizations of *strategies for navigation*, differing in cartographic and linguistic information (Miller and Santoni, 1986; Silverman and Eals, 1992; Galea and Kimura, 1993; Eals and Silverman, 1994; Denis, 1997; Dabbs et al., 1998; Montello et al., 1999; Lawton, 2001). The most comprehensive distinction divides navigation strategies into hippocampus-dependent place strategies and caudate-dependent response strategies (Packard and McGaugh, 1992; McDonald and White, 1994; Bohbot et al., 2012). This distinction originates from the field of spatial learning, meaning the manner in which the navigator acquires and receives information about the environment and navigates it.

In order to navigate through space, information about the direction the navigator is traveling (or has traveled) and distance the navigator has to scale (or has scaled) is necessary. Both, direction and distance can be represented in varying forms. Directions can be represented using an allocentric or egocentric reference frame. An allocentric reference frame is independent of the navigators’ position, e.g., cardinal directions (“north,” “east,” “south,” “west”). In real world settings distal landmarks such as the solar altitude, mountains, oceans or star constellations can also guide the allocentric reference frame (Epstein et al., 2017). An egocentric reference frame uses the navigators’ position and direction in the environment to refer to directions (“right,” “left,” “straight ahead,” “backward/behind”). When depicting the allocentric reference frame on a chess board, the goal has fixed coordinates while the navigator adjusts his/her coordinates when moving across the board [e.g., “to get to the king on e8 (goal), I have to move my bishop from f1 to e2 to h5 to e8”]. The opposite is true for the egocentric reference frame. The relative position of the goal changes, while the navigators reference frame stays the same [“to get to the king (goal), I have to move forward with my queen, make a right, then left turn and move forward”–when taking the first person view of the queen, initially the king is straight ahead, when moving right, the king is on the left of her, when moving back left the king is straight ahead again]. The differences between allocentric and egocentric directions is the viewing perspective of the navigator.

Distances on the other hand, can be described metrically or topographically. Metric distance descriptions are absolute, precise and often described in Euclidean-terms (e.g., “27.5 m” or in chess “9 squares”). These metrics can be applied between landmarks in the environment or between the navigator and landmarks. Topographic distance descriptions on the other hand are less precise than the metric scale, refer to the relative positions of landmarks in the environment and are therefore often phrased in landmark-terms (“next to the tree” or in chess “next to the queen and bishop”). A topographic strategy is also described as being less flexible, more sequential and therefore harder to error-correct than the metric strategy (Lawton, 1996; Saucier et al., 2002).

The perception of directions (or perspective, allocentric vs. egocentric) and distances (or strategy, Euclidean vs. landmark-based) during navigation has been discussed in terms of sex differences. On average, men seem to prefer an allocentric perspective and Euclidian strategy, whereas women seem to prefer an egocentric perspective and landmark based strategy (Galea and Kimura, 1993; Dabbs et al., 1998; Saucier et al., 2002; Chen et al., 2009). These differences in navigation strategies have been suggested to explain, why men, on average, make fewer navigational errors and are faster than women in goal-directed navigation (Galea and Kimura, 1993; Astur et al., 1998; Moffat et al., 1998; van Gerven et al., 2012; Scheuringer and Pletzer, 2017). Indeed, sex differences seem to be reduced or even reversed, when more landmark-information is available in the environment (Andersen et al., 2012) or when directions are phrased in egocentric and landmark-based terms (Saucier et al., 2002; Harris et al., 2019). Nevertheless, better performance with the landmark-based strategy compared to the Euclidian strategy has been reported irrespective of participant’s sex (Sandstrom et al., 1998; Saucier et al., 2002; Scheuringer and Pletzer, 2017; Harris et al., 2019).

A certain variability in the results regarding sex differences during navigation has been attributed to the dimensionality of the task (Neubauer et al., 2010). Virtual navigation started with schematic renditions in 2D-environments, which evolved into 3D-environments and now can be studied in full immersion in virtual reality. When comparing sex differences in 3D and 2D navigation tasks, men outperformed women in 3D navigation, whereas women made fewer mistakes in the 2D task (Galea and Kimura, 1993; Lawton and Morrin, 1999; Waller et al., 2001; Saucier et al., 2002; Andreano and Cahill, 2009). This lead to the conclusion that sex differences arise in situations of higher cognitive demand (Coluccia and Louse, 2004; Forcano et al., 2009). However, a variety of differences relevant to navigation strategies and cognitive processes underlying navigation are noteworthy when comparing 3D to 2D navigation.

2D navigation task are usually depicted in a bird’s eye view, giving the person navigating an overview of the whole environment. There is no immergence in the environment, which is comparable to looking at a chess board. As a result, when navigating by egocentric directions in a 2D-environment, the confound of mental rotation has to be considered. If the environment does not rotate when the participant takes a turn (like e.g., in Scheuringer and Pletzer, 2017), a right turn when facing downward requires a left response. As mental rotation is a cognitive domain with strong sex differences favoring males (Andreano and Cahill, 2009), an egocentric perspective may be less beneficial for women in 2D navigation than 3D navigation. This may reduce sex differences in perspective taking and thus sex differences in performance. Indeed, sex differences in allocentric vs. egocentric perspective taking were more pronounced in a 3D navigation task (Harris et al., 2019) than a 2D navigation task (Scheuringer and Pletzer, 2017).

Virtual 3D environments offer more realism and immergence compared to 2D environments, since the navigator is able to have a first-person view of the environment. The mental rotation confound can be omitted, since the cardinal direction adapts to the direction the navigator is facing. The most realistic scenario for studying navigation can be obtained *via* virtual reality with a head mounted display (HMD), given that scaling distances or heights can also be represented more realistically from a first-person view immerged in the virtual world. With the increased use of virtual 3D environments during navigation, it has been discussed, whether video gaming experience plays a role for sex differences in navigation. Men tend to play more video games, which in turn relates to more experience with virtual environments, compared to women (Terlecki and Newcombe, 2005; Quaiser-Pohl et al., 2006; Terlecki et al., 2011). However, recent studies demonstrate that sex differences in video gaming experience do not mediate the sex differences observed during virtual navigation (Harris et al., 2019; van Dun et al., 2021). Accordingly, when video gaming experience is controlled and the 3D navigation task constructed in a way, that advantages due to video gaming experience are minimal, virtual navigation allows us to create similar conditions to real-life navigation, while controlling various aspects about the environment. Using a 3D virtual navigation task, Harris et al. (2019) were able to show that sex differences in navigation strategy (Euclidian vs. landmark-based strategy) were most pronounced during allocentric navigation. Thus, the allocentric perspective appears to be most suitable to detect sex differences in navigation strategies.

One method to assess navigation strategy beyond performance measures during different instructions, is eye-tracking, i.e., the assessment of eye fixations and eye movement during a cognitive task. Given that eye fixations are correlated to the focus of attention (Groner and Groner, 1989), the duration and frequency spent fixating on certain aspects of the environment, reflect the amount of cognitive resources allocated to processing this type of information (Greef et al., 2009). Several studies assess sex differences in gaze behavior during navigation (Miyahira et al., 2000a,b; Campagne et al., 2005; Mueller et al., 2008a; Cazzato et al., 2010; Andersen et al., 2012). An increased fixation duration, pupil dilation and decreased performance during a Morris water maze has been demonstrated by Mueller et al. (2008a) in women compared to men, who explored the environment more and therefore had decreased fixation periods. The preferred landmark strategy for women led investigators to believe that women would look at landmarks more frequently and longer than men (Andersen et al., 2012), but as the number of landmarks in an environment increases, so does the time spent looking at landmarks equally for men and women (Andersen et al., 2012). Accordingly, the gaze behavior of men and women has been implicated in differential strategy use between men and women, but so far, the association remains elusive. While much attention has been paid to sex differences in the fixations on landmarks in the environment, fixations on other aspects of the environment relevant to navigation strategies have not been investigated. For instance, distal landmarks are required for allocentric navigation. Accordingly, it can be expected, that landmarks and objects focused by men are at a greater distance from the navigator than landmarks and objects focused by women. However, gaze distance has not been assessed by previous eye-tracking studies on navigation. Likewise, Euclidian distances may be estimated by focusing on the floor of the environment, yet no study has considered sex differences in fixations on the floor. In the present study, we therefore assess eye-fixations on various aspects of the environment, as well as their distance to the navigator, in men and women, while simultaneously manipulating strategy use *via* different instructions using a virtual reality adaptation of a 3D-navigation task, for which differential strategy use in men and women was previously demonstrated (Harris et al., 2019).

Accordingly, we hypothesize that:

(i) Men complete the navigation task faster than women.
(ii) Irrespective of instructions, women gaze longer at landmarks than men, while men gaze longer on the floor and the walls of the environment (representing distal landmarks).
(iii) Irrespective of instructions, gaze distance is shorter in women than in men.
(iv) Sex difference in performance and eye-movements between men and women are larger when instructions require an Euclidian strategy compared to a landmark-based strategy.

In addition, given that sex differences in cognitive tasks are often viewed as modulated by hormonal effects (e.g., Peragine et al., 2020), hormonal associations to navigation strategies are an interesting field of study. For instance, it has been suggested that sex differences in spatial abilities are more pronounced during the luteal cycle phase in women, when progesterone levels peak and estradiol levels are moderately high (e.g., Hampson, 1990). While previous studies found no association of estradiol or progesterone to overall navigation performance (Scheuringer and Pletzer, 2017; Harris et al., 2019), interesting associations to navigation strategies were observed. Hussain et al. (2016) observed a stronger use of spatial strategies during the luteal cycle phase in a 3D spatial learning task. Spatial strategies are characterized by an allocentric perspective, but increased use of landmarks. Dissociating perspective and strategy in a 2D wayfinding task, Scheuringer and Pletzer (2017) also found increased accuracy with landmark-based instructions during the luteal cycle phase, but higher levels of progesterone related to an increased accuracy for the egocentric compared to the allocentric perspective. In summary, it appears that menstrual cycle control is relevant when studying sex differences in navigation strategies with the luteal phase appearing most sensitive to sex differences in landmark-based strategies.

On the other hand, it has often been speculated that the male advantage during navigation may be the result of higher testosterone levels in men. Indeed, circulating testosterone levels have sometimes been related to improved performance in spatial tasks (Gordon et al., 1986; Silverman et al., 1999; Hausmann et al., 2000, 2009; Aleman et al., 2004; Hooven et al., 2004; Driscoll et al., 2005; Burkitt et al., 2007; Yang et al., 2007; Mueller et al., 2008b; but see: McKeever et al., 1987; Puts et al., 2010). Accordingly, the question arises, whether testosterone facilitates an allocentric perspective and Euclidian strategy. Only few studies have focused on this question so far, yielding non-significant associations (Harris et al., 2019). However, previous studies in related tasks suggest that eye-movements, which have a more direct link to attentional processes (e.g., Shepherd et al., 1986; Theeuwes et al., 2009; Orquin and Loose, 2013) may be more susceptible to hormonal influences than purely behavioral measures like reaction time (Schulte et al., 2020). Accordingly, we explore, whether the sex hormones estradiol, progesterone and testosterone, mediate or moderate the sex differences in performance, fixation duration and gaze distance.

## Materials and Methods

### Participants

A total of 86 healthy participants, aged 18–34 years, 41 men and 45 women during their luteal menstrual cycle phase were recruited for this study. Exclusion criteria for participants were physical, endocrine or mental illness, medication and left-handedness, as well as hormonal contraception in women. Furthermore, all participants had to either have sufficient eyesight or wear soft contact lenses, due to the physical restrictions of the virtual reality goggles. Women were required to have a regular (Fehring et al., 2006) menstrual cycle ranging from 21 to 35 days. An increased use of landmark information during the high-hormone mid-luteal phase was demonstrated in previous studies concerning navigation (Hussain et al., 2016; Scheuringer and Pletzer, 2017). Accordingly, this cycle phase was chosen to evaluate sex differences in the present study. The mid-luteal phase was determined based on self-reports of the participants of the onset of their last three menses and resulting cycle length. Test sessions were scheduled 3–10 days before the expected onset of next menstruation, which was confirmed by follow-up reports.

Seven participants were excluded due to nausea (six women and one men). Four participants were excluded due to deviations of progesterone levels (for details see hormonal analysis, Harris et al., 2019), indicating anovulatory cycles (Seifert-Klauss, 2020). Accordingly, the final sample consisted of 75 participants, 39 men (mean age: 23.13; SD: 3.38) and 36 women (mean age: 24.06; SD: 3.54), with an average cycle length of 29.31 days (SD: 2.73).

All participants had received a minimum of 8 years of secondary education, and had passed general qualification for university entrance. Age [*t*_(73)_ = −1.16, *p* = 0.25, *d* = 0.27] and IQ [*t*_(73)_ = 1.70, *p* = 0.09, *d* = 0.39] did not differ significantly between men and women. However, video gaming experience [*t*_(73)_ = 2.75, *p* = 0.007, *d* = 0.64] and perceived video gaming skill [*t*_(73)_ = 3.00, *p* = 0.004, *d* = 0.69] differed significantly between men and women. Men had played more video games than women (1.69 vs. 0.72 of possible 6) and had subjectively higher skill level at playing video games (3.77 vs. 2.00 of possible 7).

### Ethics Statement

All participants gave their informed written consent to participate in the study. The methods used conformed to the Code of Ethics of the World Medical Association (Declaration of Helsinki). The study was approved by the local ethics committee.

### Navigation Task

The navigation task used in the present study was an adaptation of the 3D navigation task developed by Harris et al. (2019). The current version was specifically adapted for use in virtual reality (VR). Ten virtual environments and two training environments were created with the Unreal Engine 4 Version 18.3 and were presented *via* the VR headset HTC-Vive. Each environment consisted of 100 squares (10 × 10 matrix) representing the floor of the environment. The floor squares were arranged in alternating light brown und light green chessboard checker pattern. Each square was populated with one of ten real-life landmarks (tree, bridge, stairs, house, church, bench, boulder, streetlight, fence, flowers), in different orders. Importantly, each landmark only occurred once in each row and column. As surrogates for distal landmarks semi-transparent walls were implemented as physical boundaries around each environment.

**FIGURE 1:**
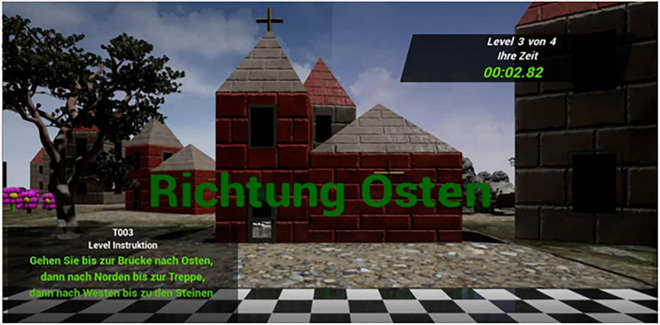
Navigation environment from the view of the participant on the start field. The participant can see the cardinal direction they are facing (in this case east) as well as three lines of directions phrased from an allocentric perspective and – in this case – landmark-based (translation: Go east to until you reach the bridge, then go north until you reach the stairs, then go west until you reach the rocks).

After the two training levels, the participants completed the ten task levels. At the beginning of each level participants were positioned on a starting square connected to the matrix of the environment. The position of the starting square varied in location, meaning it was either located on the north, east, south or west border of the matrices, counterbalanced across the 12 environments. All levels started with a countdown (“three, two, one, go”), followed by information about the cardinal directions participants were initially facing (compare Figure 1). Wearing the virtual reality googles and using the arrow keys of a keyboard for motion control (forward and backward for locomotion; left and right arrow key or head rotation to change orientation), the participants navigated according to the instructions given to them. Their task was to reach a target location (goal) following a path indicated by three lines of directions. Each path encompassed 15 squares and 2 turns, only moving forward. The movement patterns were either U-shaped (two left or right turns) or Z-shaped (one left and one right turn). The participants were able to summon and consult the directions anytime upon pressing the 0-button of the number pad next to the arrow keys on a standard keyboard. When releasing the 0-button the directions disappeared. All directions were phrased in allocentric terms, given that this perspective was most sensitive to sex differences in navigation strategy (Harris et al., 2019). Navigation strategy was varied in pseudo-randomized order. In five task levels and one training level each, directions were phrased in Euclidian terms or landmark terms, respectively (Euclidian: “go east for 4 blocks”; landmark based: “go east until you reach the tree”).

Participants were only able to move to the next level upon completion of the current level, i.e., when reaching the goal. Reaching the goal was confirmed *via* pressing the space bar. Accordingly, the performance measure for the navigation task was the navigation time in seconds, from start (“go”) to completion (correct confirmation *via* space bar), while accuracy was a prerequisite to complete the task.

### Eye-Tracking

The aGlass DKII VR-Eye-Tracking add-on by 7invensun for the HTC Vive was used to record eye-movement. The trackers were built into the HTC Vive, in front of both screens with a field of view of >110°. The tracking speed is stated to be 100–120 Hz with a latency of <5 ms and accuracy of <0.5° (aGlass DKII User manual, 2021). The fixation point was translated into the 3D environment and the following measures were recorded upon collision with an object in the 3D environment: (i) duration of the collision (= absolute fixation duration), and (ii) distance between first-person and collision object. Collision objects were categorized into (i) landmarks, (ii) walls surrounding the environment (representing distal landmarks), (iii) floor, and (iv) sky above the environment. The relative fixation duration was calculated as percentage of the navigation time, by dividing the absolute fixation duration by navigation time (NT) multiplied by 100 for each item. The distance of fixations was measured in Unreal Units (uu) with a conversion rate of 1,000 uu = 10 m. The size of each map was about 12,000 uu^2^ (120 m^2^), 1,200 uu^2^ per square and the maximum walking speed was set to 1,200 uu/s.

### Procedure

All testing took place between 8:30 a.m. and 5:30 p.m. at the computer lab at the University of Salzburg and took about 60–90 min to complete. Participants were asked not to eat, drink or smoke prior to testing. After rinsing out their mouths for the saliva sample, participants filled out the informed consent and the exclusion criteria and health screening questionnaires. Information about the head mounted VR-googles and motion sickness were given and for women, a hormone questionnaire was filled out, followed by the retrieval of the first saliva sample. After the completion of a mental rotation task the second saliva sample was taken and the VR-headset, eye-tracking and navigation task were explained verbally. An overview of a training level was presented and the objective of the navigation task and the different objects were described. Participants were seated in an office chair with a wireless standard keyboard in their laps for motion control. The VR-Headset and eye-tracker were calibrated for each participant individually, adjusting the lens distance and using a nine-point dial, fixating points without moving their heads (aGlass DKII User manual, 2021). Afterward, participants could adjust to the VR-glasses, headset and the instructions in the two training levels. Participants completed the training levels, as well as the navigation task and were asked for the last saliva sample. Finally, all participants answered questionnaires pertaining to video gaming experience, gender identity and for women a premenstrual syndrome questionnaire (PSST, premenstrual syndrome screening tool). The ten-item APM screening (advanced progressive matrices) as implemented in the Vienna Test System was chosen to ensure no substantial differences in IQ and sufficient abilities to recognize and process new patterns during the navigation task. In the end participants were debriefed and received either course credit or 10€ for participating.

### Hormonal Analyses

Three saliva samples of each participant were collected (compare the “Procedure” section), stored at −20°C and centrifuged twice at 3,000 rpm for 15 and 10 min, respectively, prior to hormone assessment. In order to assess average hormone concentrations over the whole session, the three samples of each participant were pooled before hormone analysis, thereby ensuring the reliability of hormone assessment and controlling for fluctuations related to salivary production. Using salivary ELISA kits by DeMediTec, testosterone, 17β-estradiol, and progesterone were assessed from the pooled sample of each participant. Deviations of hormone values of the participants by more than three standard deviations from the group mean were excluded. Further, it was expected that progesterone values of women fall within a normal range for the luteal cycle phase. First, we established this range for our laboratory based on an unrelated sample of 60 women tested with the DeMediTec salivary progesterone ELISA kit in three menstrual cycle phases, as recommended by the DeMediTec kit instructions (compare Pletzer et al., 2018). Progesterone levels in all women were higher during the luteal phase compared to the other cycle phases. All women displayed a luteal progesterone value above 48 pg/ml, although with considerable variation. Due to the assay sensitivity of 5 pg/ml a progesterone cutoff of 43 pg/ml was established for inclusion in a luteal phase sample. In the current sample, four women were excluded due to progesterone levels below 43 pg/ml.

### Statistical Analyses

Statistical analysis was performed using R 3.6.3 in RStudio 1.2.5033. Prior to analysis, outliers (defined as >3 standard deviations above the mean) among navigation time, gaze duration or gaze distance were excluded. Navigation time (in seconds), relative fixation duration (in % NT) for landmarks, floor, sky and wall, as well as distance of fixations (in uu) for landmarks, floor, sky and wall, were analyzed in the context of linear mixed effects models (lmes) using the lmer function of lme4 package (Version 1.1-21). In all models, the participant number was modeled as a random factor. The following models were evaluated:

First, we addressed sex differences in the dependent variables and their modulation by strategy by introducing the interactive effect of sex × instruction as a fixed effect in the model, while controlling for age, IQ and video gaming experience as continuous covariates (e.g., NT ∼ 1| PNr + sex × instruction + age + IQ + gaming experience).

Second, sex hormone influences on the dependent variables were addressed by the following procedure. Since none of the lmes showed a significant sex × instruction interaction or significant effects of age, IQ or video gaming experience, the data of the different instructions were merged for each participant and further analyzed in the context of linear models. In a first step, the sex × hormone interaction was entered into the models (e.g., NT ∼ sex × hormone) to assess moderator effects of sex on the sex hormone associations. If the sex × hormone interaction was non-significant, suggesting no moderation of sex hormone influences by sex, the interaction was dropped. In a second step, we thus analyzed the hormonal associations while controlling for the effect of sex (e.g., NT ∼ sex + hormone). In these models we further evaluated mediatory influences of sex hormones, by assessing, if previously significant effects of sex remained significant after entering the hormone into the model.

In all models, both, the dependent and continuous independent variables were z-standardized using the scale function. Therefore, the coefficients b of fixed effects in the models represent a standardized effect size based on standard deviations, similar to Cohen’s d. Analyses on gaze duration and gaze distance were FDR-corrected for the four different objects assessed (landmarks, floor, sky, walls).

## Results

### Sex Differences in Navigation Time

**FIGURE 2:**
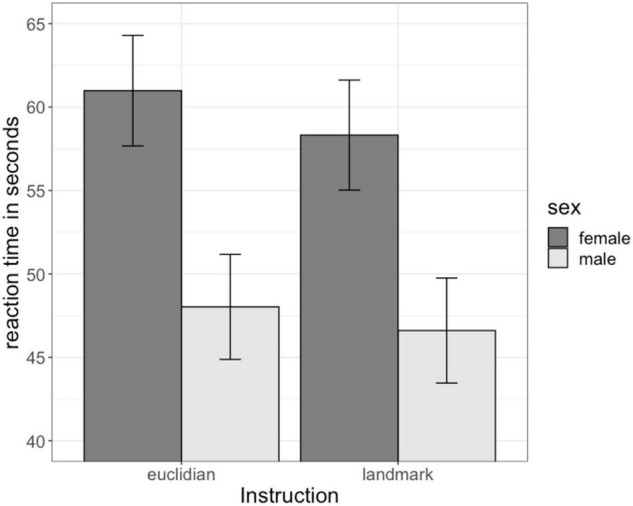
Sex differences in navigation time. Men navigated significantly faster than women irrespective of the instruction. Instruction had no significant effect on navigation time.

The main effect of sex on navigation time was significant [*b* = 0.29, SE_*b*_ = 0.15, *t*_(70)_ = 2.00, *p* <0.05, Figure 2]. Men showed a faster navigation time (48.96 s, SE: 2.84) compared to women (57.82 s, SE: 3.00). Surprisingly, the main effect of instruction [*b* = −0.05, SE_*b*_ = 0.09, *t*_(626)_ = −0.48, *p* = 0.63; Euclidian: 54.17 s, SE: 2.05; Landmark: 52.1 s; SE: 2.05], as well as the sex × instruction interaction [*b* = −0.04, SE_*b*_ = 0.13, *t*_(626)_ = −0.29, *p* = 0.77] were not significant. The factors age, IQ and video gaming were also non-significant [all | b| < 0.13, all SEb < 0.07, all | t_(70)_|<1.94, all *p* > 0.05].

### Sex Differences in Gaze Duration

Relative fixation duration describes what percentage of their time in an environment, participants spent looking at various objects in the environment, i.e., the landmarks, the floor, the sky or the walls surrounding the environment. Participants spent the majority of their time fixating the floor (49%, SD = 26%), followed by fixations on landmarks (31%, SD = 17%) and fixations on the sky (18%, SD = 25%). Only a very small percentage of the time was spent fixating the walls of the environment (0.7%, SD = 1%).

There were no significant differences between men and women regarding the fixation durations on landmarks [*b* = 0.08, SE_*b*_ = 0.18, *t*_(70)_ = 0.43, p_*FDR*_ = 0.87] and landmark-based instructions did not elicit significantly longer fixations on landmarks than Euclidian instructions [*b* = −0.002, SE_*b*_ = 0.08, *t*_(626)_ = −0.03, p_*FDR*_ = 0.98]. However, significant differences due to sex and instruction did arise regarding other fixation points in the environment.

For fixations on the walls, the main effect of sex was not significant [*b* = 0.02, SE_*b*_ = 0.12, *t*_(70)_ = 0.17, p_*FDR*_ = 0.87], however the main effect of instruction was significant [*b* = 0.46, SE_*b*_ = 0.10, *t*_(626)_ = 4.74, p_*FDR*_ < 0.001]. Fixations on the walls were longer with landmark-based instructions compared to the Euclidian instructions.

**FIGURE 3:**
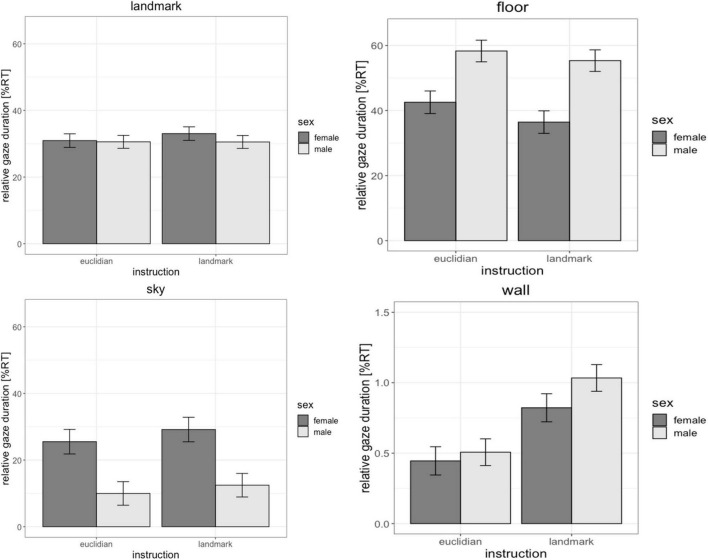
Effects of sex and instruction on the duration of fixations on various objects in the environment. Men and women did not differ in the duration of their fixations on landmarks or the walls of the environment. However, women looked longer at the sky, while men looked longer at the floor. Fixations on the walls of the environment were longer with the landmark instruction compared to the Euclidian instruction. NT, navigation time.

For fixations on the floor and sky, the main effects of sex was significant [floor: *b* = −0.60, SE_*b*_ = 0.20, *t*_(70)_ = −2.95, p_*FDR*_ = 0.018; sky: *b* = 0.56, SE_*b*_ = 0.22, *t*_(70)_ = 2.59, p_*FDR*_ = 0.024], while the main effect of instruction was not significant [wall: *b* = −0.12, SE_*b*_ = 0.06, *t*_(626)_ = −1.93, p_*FDR*_ = 0.072; sky: *b* = 0.10, SE_*b*_ = 0.11, *t*_(626)_ = 1.47, p_*FDR*_ = 0.072]. Women fixated the sky longer than men, while men fixated the floor longer than women (Figure 3). The sex × instruction interaction, as well as the factors age, IQ and video gaming skill were non-significant in all models [all | b| < 0.15, all SE_*b*_ < 0.14, all | t| _(626)_ < 1.56, all *p* > 0.07, p_*FDR*_ > 0.104].

### Sex Differences in Fixation Distance

**FIGURE 4:**
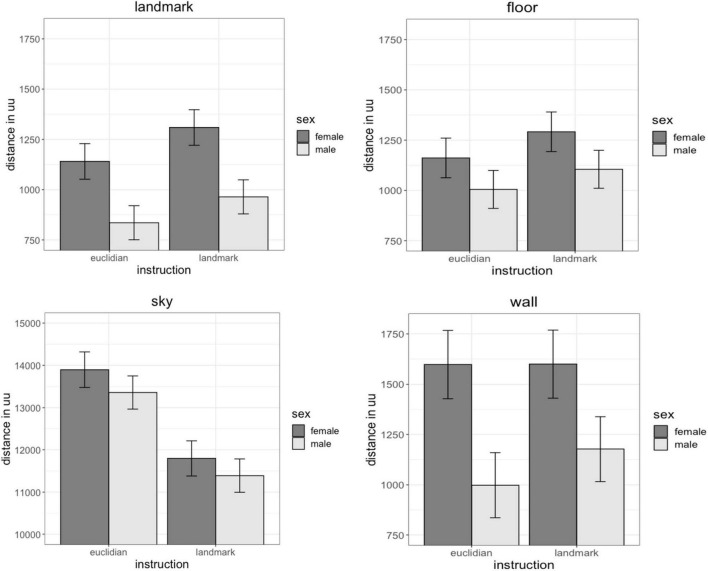
Effects of sex and instruction on gaze distance. Women fixated landmarks, floors and walls further away than men. Landmark-instruction elicited fixations further away for landmarks and floor, but closer by for the sky than Euclidian instructions.

Fixation distance describes how far away the objects were on average that participants fixated their gaze on. Landmark-fixations were closest (1,042 uu, SD = 660 uu), followed by floor fixations (1,130 uu, SD = 730 uu) and wall fixations (1,328 uu, SD = 1,504 uu). Sky fixations showed the largest distance (12,593 uu, SD = 5,447 uu). For distance of fixations on landmarks and walls the main effect of sex was significant [landmarks: *b* = 0.47, SE_*b*_ = 0.20, *t*_(70)_ = 2.34, p_*FDR*_ = 0.04; walls: *b* = 0.41, SE_*b*_ = 0.17, *t*_(70)_ = 2.44, p_*FDR*_ = 0.04]. Irrespective of the object fixated, women’s fixations were further away than men’s fixations (Figure 4). No sex differences were observed in the distance of fixations on the floor [*b* = 0.27, SE_*b*_ = 0.20, *t*_(70)_ = 1.36, p_*FDR*_ = 0.24] and sky [*b* = 0.11, SE_*b*_ = 0.11, *t*_(70)_ = 1.01, p_*FDR*_ = 0.32]. The main effect of instruction was significant for the distance of fixations on landmarks, floor and sky [landmarks: *b* = 0.19, SE_*b*_ = 0.07, *t*_(626)_ = 2.89, p_*FDR*_ = 0.008; floor: *b* = 0.14, SE_*b*_ = 0.07, *t*_(626)_ = 2.00, p_*FDR*_ = 0.06; sky: *b* = −0.36., SE_*b*_ = 0.10, *t*_(626)_ = −3.54, p_*FDR*_ = 0.002]. Landmark and floor fixations were further away, while sky fixations were closer for landmark-based compared to Euclidian instructions. No difference in gaze distance between landmark-based and Euclidian instructions was observed for fixations on the wall [*b* = 0.12, SE_*b*_ = 0.09, *t*_(626)_ = 1.40, p_*FDR*_ = 0.16]. The sex × instruction interaction, as well as the factors age, IQ and video gaming skill were non-significant in all models [all | b| < 0.16, all SE_*b*_ < 0.15, all | t| _(457)_ < −1.05, all *p* > 0.292].

### Predicting Navigation Time by Gaze Distance

In order to understand, how eye gaze behavior contributed to navigation performance, we assessed associations between gaze distance and navigation time. Navigation time could be predicted by gaze distance for the wall and sky [wall: *b* = 0.10, SE_*b*_ = 0.04, *t*_(624)_ = 2.34, *p* = 0.02; sky: *b* = 0.13, SE_*b*_ = 0.03, *t*_(624)_ = 3.96, *p* = 0.0001], but not for the floor and landmarks [landmarks: *b* = 0.007, SE_*b*_ = 0.05, *t*_(624)_ = 0.14, *p* = 0.89; floor: *b* = −0.04, SE_*b*_ = 0.05, *t*_(624)_ = −0.79, *p* = 0.43]. Reaction times were longer with greater gaze distance for the wall and sky. When entering gaze distance as additional predictor to the model exploring sex differences in navigation time, the sex difference remained significant.

### Sex Hormones and Navigation Time

There were no significant associations between sex hormones and navigation time, while controlling for sex. However, entering testosterone in the model rendered the sex difference in navigation time non-significant, suggesting a partial mediation of the sex difference by testosterone levels.

### Sex Hormones and Gaze Duration

**FIGURE 5:**
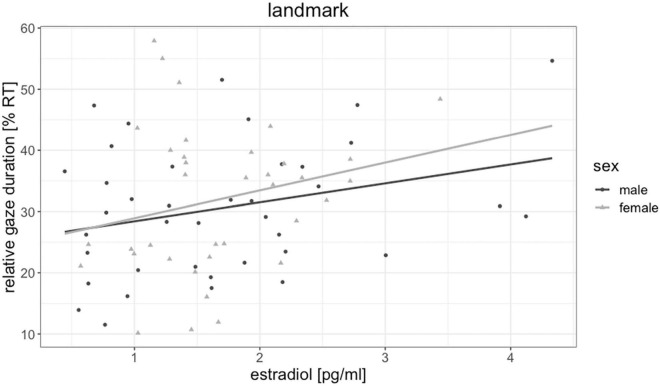
Association of estradiol to landmark gaze duration in men and women. The higher participants’ estradiol levels, the more time they spent looking at landmarks.

**FIGURE 6:**
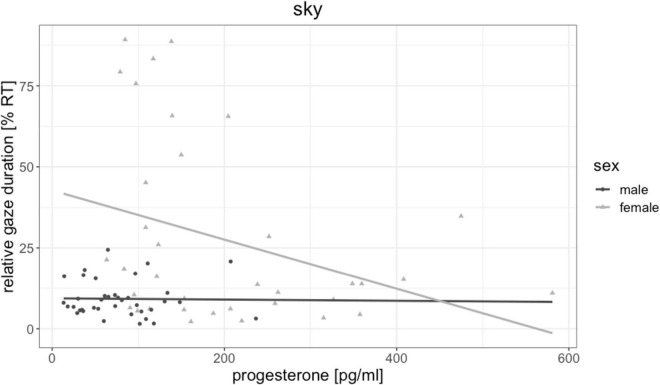
Association of progesterone to sky gaze duration. The higher participants’ progesterone levels, the less time they spent gazing at the sky. This association was driven by female participants.

Testosterone was not significantly related to fixation duration of any object [all | b| < 0.19, all SE_*b*_ < 0.18, all | t_(72)_| < 0.87, all *p* > 0.303] and did not mediate the sex difference in floor and sky gaze duration. Estradiol was by trend related to fixation durations on landmarks [*b* = 0.26, SE_*b*_ = 0.11, *t*_(72)_ = 2.24, *p* = 0.028, p_*FDR*_ = 0.111, Figure 5], but not to fixation duration on any other object. The higher participants estradiol levels, the longer were their fixations on landmarks. Progesterone was significantly negatively related to fixation durations on the sky [*b* = −0.33, SE_*b*_ = 0.12, *t*_(71)_ = −2.70, *p* = 0.009, p_*FDR*_ = 0.036, Figure 6], but not to fixation duration on any other object. The higher participant’s progesterone levels, the less time they spent looking at the sky.

### Sex Hormones and Fixation Distance

**FIGURE 7:**
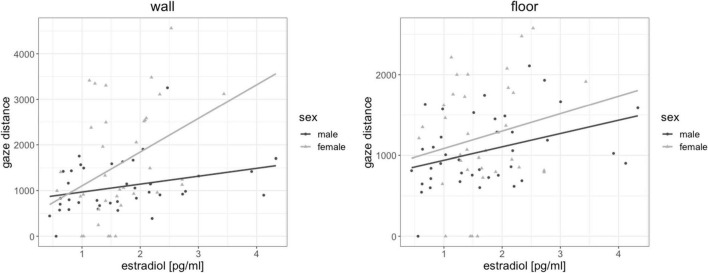
Association of estradiol to gaze distance. The higher participant’s estradiol levels, the further away were the objects they fixated on.

Testosterone and progesterone were not related to gaze distance for any object [all | b| < 0.14, all SE_*b*_ < 0.17, all | t| _(72)_ < −0.796, all *p* > 0.429]. However, when testosterone was included in the model, sex differences in gaze distance disappeared for all objects, suggesting a partial mediation of sex differences in gaze distance by testosterone. When progesterone was included in the model, sex differences in gaze distance disappeared for wall fixations, also suggesting a partial mediation of gaze distance by progesterone. Estradiol was significantly related to gaze distance in wall fixations [*b* = 0.28, SE_*b*_ = 0.11, *t*_(72)_ = 2.57, p_*FDR*_ = 0.048] and floor fixations [*b* = 0.26, SE_*b*_ = 0.11, *t*_(72)_ = 2.31, p_*FDR*_ = 0.048]. The higher participant’s estradiol levels, the further away were the objects they fixated on (Figure 7).

## Discussion

The present study aimed to investigate whether sex differences in navigation strategy were reflected in eye movements during a virtual navigation task and whether these sex differences were moderated or mediated by the sex hormones estradiol, progesterone and testosterone,. Based on previous work we expected faster navigation in men compared to women, longer fixations on landmarks, but shorter fixations on the floor and the walls of the environment (distal landmarks) in women compared to men, as well as a larger gaze distance in men compared to women. We furthermore expected all sex differences to be larger with Euclidian compared to landmark-based instructions.

As expected, navigation times were faster in men compared to women, which is in line with a large number of studies demonstrating sex differences in navigation performance (Galea and Kimura, 1993; Astur et al., 1998; Moffat et al., 1998; Saucier et al., 2002; Andreano and Cahill, 2009; van Gerven et al., 2012; Scheuringer and Pletzer, 2017). Please note, however, that the task may have been subject to sex differences in speed-accuracy trade-offs (Bianco et al., 2020), given that maximum accuracy was required to complete the task.

Also in line with our hypotheses, men showed longer fixations on the floor compared to women, which indicates a preference for a Euclidian based strategy, where distances need to be estimated in Euclidian terms. However, all sex differences were irrespective of the instructions provided. Regarding navigation time, this suggests, that contrary to previous navigation tasks (Saucier et al., 2002; Harris et al., 2019), in the virtual environment women did not have an advantage with landmark-based instructions. Regarding eye fixations, the lack of a modulation by instruction suggests that the attentional processes guiding eye gaze in the virtual environment are not modulated by the phrasing of the instructions. Thus, the fixation durations may be more reflective of the intrinsic strategy preference of the participant rather than the strategy required by the instructions.

Not in line with our hypotheses, and contrary to a number of previous works demonstrating a preference for landmark-information in women (Montello et al., 1999; Coluccia and Louse, 2004; Andersen et al., 2012), is the result that men and women did not differ in their fixation durations on local or distal landmarks (walls) in the environment. This is, however, in accordance with the behavioral finding, that navigation time in women did not improve with landmark-based instructions. Taken together, these results suggest that in this particular implementation of the navigation task, women were unable to optimally utilize the landmark-information available, in order to improve their navigation performance. It is possible that the novelty and unfamiliarity of the virtual environment played a role in that respect.

While visually, the VR provides a more realistic environment to other computerized navigation tasks and allows the continuous and efficient tracking of eye-movements during navigation, there is only limited proprioceptive and vestibular feedback, which provides information on head and limb position and orientation, as well as linear acceleration and rotation (Christou et al., 2016). The lack of this information can impair one’s ability to dynamically update one’s position on the cognitive map, which in turn affects performance (Chance et al., 1998; Klatzky et al., 1998; Christou et al., 2016). It is possible, that women were more affected by the lack of proprioceptive and vestibular feedback than men. Indeed, some studies demonstrate sex differences in proprioception (Hu et al., 2020) and inter-modal integration (Sigmundsson et al., 2007). If men and women are differentially affected by the limitation of the VR, the question arises, whether VR is in fact the optimal method to study sex differences in navigation.

Future studies may overcome this confound by combining the VR with different types of locomotion, i.e., either walking in place with natural head-movement (Slater et al., 1995) or a more costly and complicated alternative, with omnidirectional treadmills (Souman et al., 2008). Real walking, such as done with omnidirectional treadmills, in combination with VR-googles yielded similar results as real-world movement (Suma et al., 2007).

Differential effects of the VR environment on men and women, may also explain our results regarding gaze distance, which are contrary to our hypotheses. Women showed longer fixations on the sky and larger fixation distances across all objects compared to men. This finding is in fact opposite to previous results, suggesting that men screen larger parts of the environment and focus stronger on more distal landmarks, due to their preference for allocentric navigation (Galea and Kimura, 1993; Lawton, 1996; Saucier et al., 2002). More importantly, larger gaze distance seems to be related to longer navigation times and may thus be partly responsible for the sex difference in navigation performance, although the sex difference did remain significant when controlling for gaze distance in the model. One possible explanation for women directing their gaze further away could be motion sickness, which is a common problem with VR. While subjects were specifically recruited for a certain tolerance regarding motion sickness, we did observe that more women than men had to discontinue the experiment due to severe motion sickness (compare Participant section). This is in line with previous works demonstrating that women are more susceptible to motion sickness than men (Munafo et al., 2017).

To obtain some tentative information on this issue, we did assess the motion sickness subjects experienced during the experiment, in a follow-up survey. 32 participants (16 men, 16 women) did return this survey. However, there was only a non-significant trend toward stronger motion sickness in women compared to men [*t*_(29)_ = −1.58, *p* = 0.13], motion sickness did not relate to navigation time, sky gaze duration or gaze distance and did not mediate the sex differences observed in any of the variables. While this information does only apply to a part of the sample and it is unclear whether it generalizes to the whole sample, these data suggest, that motion sickness was not entirely responsible for the sex differences we observed in gaze distance. It is, however, possible that the requirement of not being prone to motion sickness in order to be able to complete the experiment, resulted in a selection bias, such that only women with a high tolerability of motion sickness were included in the experiment. If some of the factors affecting one’s predisposition toward motion sickness are the same factors affecting attentional processes during navigation (like e.g., the way proprioceptive information is utilized), it is possible that sex differences in the current study were underestimated or reversed compared to real-life navigation. However, the fact that the larger gaze distance relates to longer navigation times, suggests that this may be one of the attentional processes contributing to the performance differences between men and women.

Finally, we did explore the relationship of sex hormone levels to eye gaze behavior, though the results should be treated as tentative due to the correlational nature of the analyses. With respect to estradiol and progesterone assessment, the fact that all women were tested in the luteal cycle phase represents a potential limitation, since the scheduling of test sessions around the individual progesterone peak limits the variability of progesterone levels. Furthermore, the analyses employed are focused on inter-individual variability in sex hormone levels, rather than intra-individual fluctuations, as have been assessed in previous longitudinal menstrual cycle studies (Hussain et al., 2016; Scheuringer and Pletzer, 2017). However, progesterone actions may be functionally different between the follicular and luteal cycle phase, given that the pre-ovulatory rise in estradiol primes the expression of progesterone receptors. Thus, while the lack of an intra-individual comparison to the follicular phase limits the interpretability of our results, the brain is likely most sensitive to progesterone actions in the luteal cycle phase. We would like to point out, however, that such cross-sectional correlational approaches focusing on the inter-individual hormonal variability are quite common with respect to testosterone, even though substantial intra-individual variability in testosterone levels has been demonstrated in men (Celec et al., 2003). Accordingly, we view the following results as hypothesis-generating for future studies.

First, we observed some associations of eye gaze behavior to estradiol and progesterone irrespective of participants’ sex. Higher estradiol levels were related to longer fixations of landmarks and a larger gaze distance for walls and floors. The fact that estradiol relates to increased landmark fixation is in line with a previous study, demonstrating a menstrual cycle modulation of landmark fixations, with more landmark fixations during the luteal cycle phase, when estradiol and progesterone levels are both elevated (Hussain et al., 2016). Accordingly, our results provide a first indication, which hormone may be responsible for these changes along the menstrual cycle. One explanation for the association of estradiol to gaze distance may again be related to motion sickness, since estradiol has been discussed to increase the susceptibility for motion sickness (Ge and Huang, 2013) and directing one’s gaze further away may help to compensate for these effects. Nevertheless, future work should also explore the possibility, that estradiol shifts attention to more allocentric aspects of the environment.

Second, there is, however, some weak indication for a partial mediation by testosterone, since sex differences in navigation time and gaze distance disappear, when testosterone is controlled in the model. Nevertheless, apart from the activational effects of testosterone, other factors may contribute to these sex differences, including genetic factors, organizational effects of said hormones during brain development, and socialization (Roof, 1993; Berenbaum and Snyder, 1995; Ruble et al., 2006; Zosuls et al., 2011; Trent and Davies, 2012).

In summary, our study demonstrated sex differences in eye-movements during navigation in a virtual environment for the first time. These sex differences are in line with a preference for Euclidian information in men, while a preference for landmark information in women could not be confirmed. However, men and women may have been differentially affected by the lack of proprioceptive and vestibular information in the VR, an avenue that should be further explored in future studies.

## Data Availability Statement

The datasets presented in this study can be found in online repositories. The names of the repository/repositories and accession number(s) can be found below: https://osf.io/pc94v/.

## Ethics Statement

The studies involving human participants were reviewed and approved by the Ethics Committee at the University of Salzburg. The patients/participants provided their written informed consent to participate in this study.

## Author Contributions

TH was involved in data collection, performed the analyses, and wrote the manuscript. BP designed the study performed the analyses and wrote the manuscript. JH assisted in data collection. All authors contributed to the article and approved the submitted version.

## Conflict of Interest

The authors declare that the research was conducted in the absence of any commercial or financial relationships that could be construed as a potential conflict of interest.

## Publisher’s Note

All claims expressed in this article are solely those of the authors and do not necessarily represent those of their affiliated organizations, or those of the publisher, the editors and the reviewers. Any product that may be evaluated in this article, or claim that may be made by its manufacturer, is not guaranteed or endorsed by the publisher.
